# Genome-wide analysis and expression profile of the bZIP gene family in *Neopyropia yezoensis*

**DOI:** 10.3389/fpls.2024.1461922

**Published:** 2024-10-21

**Authors:** Xinyu Zhu, Tian Gao, Ka Bian, Chengzhen Meng, Xianghai Tang, Yunxiang Mao

**Affiliations:** Key Laboratory of Marine Genetics and Breeding (Ministry of Education), College of Marine Life Sciences, Ocean University of China, Qingdao, China

**Keywords:** *Neopyropia yezoensis*, basic leucine zipper family, phylogenetic analysis, expression profile, nitrogen stress

## Abstract

The basic leucine zipper (bZIP) family consists of conserved transcription factors which are widely present in eukaryotes and play important regulatory roles in plant growth, development, and stress responses. *Neopyropia yezoensis* is a red marine macroalga of significant economic importance; however, their bZIP family members and functions have not been systematically identified and analyzed. In the present study, the *bZIP* gene family in *Ny. yezoensis* was characterized by investigating gene structures, conserved motifs, phylogenetic relationships, chromosomal localizations, gene duplication events, cis-regulatory elements, and expression profiles. Twenty-three *Ny. yezoensis bZIP* (*NyybZIP*) genes were identified and sorted into 13 out of 30 groups, which were classified based on the bZIPs of *Ny. yezoensis* and 15 other red algae species. Phylogenetic analysis revealed that bZIP genes may have a complex evolutionary pattern in red algae. Cross-species collinearity analysis indicated that the *bZIP* genes in *Ny. yezoensis*, *Neoporphyra haitanensis*, and *Porphyra umbilicalis* are highly evolutionarily conserved. In addition, we identified four main categories of cis-elements, including development-related, light-responsive, phytohormone-responsive and stress-responsive promoter sequences in *NyybZIP* genes. Finally, RNA sequencing data and quantitative real-time PCR (qRT-PCR) showed that *NyybZIP* genes exhibited different expression patterns depending on the life stage. *NyybZIP* genes were also found to be involved in the nitrogen stress response. We thought that bZIP genes may be involved in *Ny. yezoensis* growth and development, and play a significant role in nitrogen deficiency response. Taken together, our findings provide new insights into the roles of the bZIP gene family and provide a basis for additional research into its evolutionary history and biological functions.

## 1 Introduction

A significant portion of eukaryotic genomes consists of transcription factor (TF) genes, which are predominantly categorized into a few distinct and often sizable gene families based on the type of DNA-binding domain they encode (Riechmann and Ratcliffe, 2000). In plants, TFs play crucial roles in numerous biological pathways (Wu et al., 2023). Among the eukaryotic TF gene families, the basic region-leucine zipper (bZIP) family is the most extensive and heterogeneous group [Hurst, 1995; Wang et al., 2011]. Named after its highly conserved domain of approximately 60–80 amino acids, the bZIP includes two functional regions: a basic region and a leucine zipper region (Jakoby et al., 2002). Following systematic identification and classification in *Arabidopsis thaliana* (Dröge-Laser et al., 2018), 78 bZIP family members were classified into 13 groups (designated A–M) based on the homology of the basic region, and additional conserved motifs were proposed (Dröge-Laser et al., 2018). To date, a comprehensive understanding of *bZIP* genes have been attained in several plant species, including *A. thaliana* (Dröge-Laser et al., 2018), *Manihot esculenta* (Hu et al., 2016), *Cucumis sativus* (Baloglu et al., 2014), *Triticum aestivum* (Agarwal et al., 2019), and *Neoporphyra haitanensis* (Wang et al., 2023).

The *bZIP* genes play an indispensable role in the regulation of growth, development, and abiotic stress responses (Landschulz et al., 1988). In *A. thaliana*, *AtbZIP1* plays a role in the coordination of starvation responses triggered by darkness and NO^3-^ signaling pathways (Para et al., 2014). *AtbZIP29* is expressed in proliferating tissues (Van Leene et al., 2016), and *AtbZIP59*(*PosF21*) has been functionally implicated in auxin-mediated callus development and plant regeneration processes (Xu et al., 2018). In *Capsicum annuum*, *CabZIP25* improves salt tolerance by preserving chlorophyll stability (Gai et al., 2020). In *Nelumbo nucifera*, *NnbZIP36* facilitates anthocyanin accumulation (Zhou et al., 2023), while *PpbZIP44* in *Pyrus pyrifolia* affects the sugar:acid ratio (Wang et al., 2023). In *Aquilaria sinensis*, both *AsbZIP14* and *AsbZIP41* respond to ethylene and agarwood inducers (Zhang et al., 2023). In *Rosa*, *RcbZIP17* modulates *B. cinerea* resistance using both virus-induced gene silencing (VIGS) and overexpression (OE) approaches (Li et al., 2023). However, *bZIP* genes from red algae have not been a primary focus of investigation.

Red algae (Rhodophyta) represent a highly evolved and significant branch within eukaryotic photosynthetic organisms and constitute an independent lineage that numbers over 7000 species according to AlgaeBase (http://www.algaebase.org/), placing them among the most archaic of such groups (Lopez-Bautista, 2010). Red algae are categorized into seven distinct classes: *Cyanidiophyceae*, *Bangiophyceae*, *Florideophyceae*, *Compsopogonophyceae*, *Porphyridiophyceae*, *Rhodellophyceae*, and *Stylonematophyceae*, which collectively occupy diverse ecological niches ranging from hot springs and acidic sulfur vents, to freshwater systems, deep-sea abysses, and intertidal areas. Morphologically, red algae exhibit remarkable diversity, appearing as single-celled organisms, filamentous structures, leaf-like thalli, parenchymatous blades, and tubular forms composed of a single layer of cells (Yoon et al., 2010). *Neopyropia yezoensis* belongs to *Bangiophyceae* (*Rhodophyta*), which is the most genetically variable group of red algae and a key species in the commercial cultivation of edible marine macroalgae (Wang et al., 2016).

*Ny. yezoensis* possesses a complex life cycle characterized by alternating between two distinct multicellular phases: the haploid gametophytic stage and the diploid sporophytic stage. This lifecycle is crucial for the large-scale cultivation of *Ny. yezoensis* and its subsequent establishment and growth (Blouin et al., 2011). *Ny. yezoensis* inhabits the intertidal zone and exhibits remarkable stress tolerance, highlighting its adaptability to challenging environments (Burritt et al., 2002; Nishikawa et al., 2007; Pearson et al., 2010). For example, the growth, quality, and yield of *Ny. yezoensis* responds rapidly to the level of nitrogen in seawater (Kakinuma et al., 2008; Kakinuma et al., 2016; Li ChengZe et al., 2019). These attributes make *Ny. yezoensis* an ideal model organism for elucidating the evolutionary trajectory of interconnected metabolic and regulatory pathways governing growth, reproduction, and stress adaptation (Blouin et al., 2011). The high-quality chromosomal genome of *Ny. yezoensis* has enabled comprehensive characterization of its *bZIP* genes (Wang et al., 2020). Furthermore, the analysis of existing transcriptomic datasets has facilitated an in-depth investigation into the complex molecular functions performed by these *bZIP* genes (Sun et al., 2015; Sun et al., 2019; Tang et al., 2019).

In this study, we conducted a comprehensive analysis of the *bZIP* gene family in *Ny. yezoensis* by examining the chromosomal localization, duplication events, cis-regulatory elements, gene architecture, conserved motifs, evolutionary affiliations, expression patterns, protein interaction networks, and functional annotations using gene ontology. In addition, we utilized bioinformatic techniques to identify *bZIP* gene family members in 15 additional species of red algae and performed phylogenetic analyses, thereby establishing a foundation for the *bZIP* gene family classification within red algae. Furthermore, we examined the response of *NyybZIP* genes to nitrogen stress conditions and different life cycle phases, and validated their expression patterns using transcriptomic and qRT-PCRanalyses. Taken together, our results establish a framework for future functional investigations into *NyybZIP* genes.

## 2 Materials and methods

### 2.1 Genome-wide identification and characterization of the *bZIP* gene families in *Ny. yezoensis* and other red algae

Fifteen types of red algae and one representative species each from green algae, streptophyte algae, and bryophyte were selected for bZIP protein identification. The *Cyanidioschyzon merolae*, *Chondrus crispus*, *Gracilariopsis chorda*, *Galdieria sulphuraria*, *Porphyridium purpureum*, *Ny. yezoensis*, *Porphyra umbilicalis*, *Chlamydomonas reinhardtii*, *Marchantia_polymorpha* reference genome assemblies and protein sequences were obtained from GenBank using accessions GCA_000091205.1 (Nozaki et al., 2007), GCA_000350225.2 (Janouškovec et al., 2013), GCA_003194525.1 (Lee et al., 2018), GCA_000341285.1 (Schönknecht et al., 2013), GCA_008690995.1 (Lee et al., 2019), GCA_009829735.1 (Wang et al., 2020), GCA_002049455.2 (Brawley et al., 2017), GCA_000002595.3 (Merchant et al., 2007), and GCA_037833965.1 respectively. Additional red algal and *Chara braunii* protein sequences were downloaded from the EukProt database (https://doi.org/10.6084/m9.figshare.12417881.v3) (Richter et al., 2022). *A. thaliana* bZIP protein sequences were downloaded from the Pytozome database (Goodstein et al., 2012). *No. haitanensis* bZIP protein sequences were obtained from Wang et al (Wang et al., 2023). To obtain the bZIP protein sequences from these 15 types of red algae and three representative species of Viridiplantae. the Hidden Markov model (HMM) profile (Eddy, 2004) of the bZIP_1 (PF00170) and bZIP_2 (PF07716) domain was downloaded from the Pfam database (Browse - InterPro (ebi.ac.uk)), and potential proteins were identified using local HMMER software (E-value <1e-5). Next, a local BLASTP search (E-value <1e-5) was applied to identify possible bZIPs using *A. thaliana* and *No. haitanensis* as well as the PlnTFDB database (http://plntfdb.bio.uni-potsdam.de/v3.0/). Finally, InterProScan (http://www.ebi.ac.uk/interpro/result/InterProScan/), SMART (http://smart.embl-heidelberg.de/), and CDD (https://www.ncbi.nlm.nih.gov/cdd) databases (Marchler-Bauer et al., 2017) were used for additional screening and verification of the bZIP domain. Except for *No. haitanensis*, which used the name from the original manuscript (Wang et al., 2023), the naming of bZIP proteins in other red algae was based on molecular weight. The ProtParam tool (Gasteiger et al., 2003) was used to determine the molecular weight, instability index, and theoretical isoelectric point (pI) of these red algae bZIPs. The subcellular localization of *Ny. yezoensis* bZIPs was predicted using Cell-PLoc-2 (http://www.csbio.sjtu.edu.cn/bioinf/Cell-PLoc-2/) (Chou and Shen, 2010), TBtools software (Chen et al., 2020) and the online website MEME (http://meme-suite.org/) (Bailey et al., 2006) were utilized to obtain the exon-intron structures, motifs, and visualize the chromosomal locations of the *bZIP* genes using *Ny. yezoensis* genome sequences and annotation files. The bZIP transcription factor family domains logos of *Ny. yezoensis* were generated using WebLogo (Crooks et al., 2004).

### 2.2 Phylogenetic and motif analyses of the *bZIP* gene families in *Ny. yezoensis* and other red algae

The conserved domains of bZIP protein sequences of red algae, *A. thaliana*, and other three representative species of Viridiplantae were imported into the MUSCLE program in the MEGA 11 software (Tamura et al., 2021). Then, the maximum likelihood (ML) phylogenetic tree was constructed using IQ-TREE (Minh et al., 2020) with the Ultrafast bootstrap (Hoang et al., 2018) set to 1000. ModelFinder (Kalyaanamoorthy et al., 2017) in IQ-TREE was then used to find the best-fit substitution model, and the tree figure was constructed using tvBOT (Xie et al., 2023). The same approach was used to construct a phylogenetic tree using bZIP protein sequences from 16 species of red algae and their conserved patterns in the *bZIP* gene family was determined using the online website MEME (http://meme-suite.org/) (Bailey et al., 2006).

### 2.3 Collinearity analysis of the *bZIP* gene families in *Ny. yezoensis*

Tandem and segmental duplication events of the red algal *bZIP* gene family and collinearity of the *bZIP* genes of *Ny. yezoensis* were analyzed using the One-Step MCScanX program in TBtools (Chen et al., 2020). To determine the selection pressure, the rates of nonsynonymous (Ka) and synonymous (Ks) substitutions (Zhang et al., 2006) were calculated using TBtools.

### 2.4 Cis-regulatory element analysis of the *bZIP* gene families in *Ny. yezoensis*

To predict the cis-acting elements present in *Ny. yezoensis bZIP* genes, TBtools software was used to extract the upstream 2000 bp promoter sequences of the *Ny.yezoensis bZIP* genes. Then, these sequences were submitted to PlantCARE (Lescot et al., 2002). Based on the annotated functions, the detected cis-acting regulatory elements were classified into different response types. A heat map was used to display the number of cis-acting regulatory elements detected.

### 2.5 Investigation of *NyybZIP* gene expression patterns at different life stages

To investigate the expression patterns of *NyybZIP* genes at different life stages, RNA-sequencing (RNA-Seq) data were obtained from a previous study (gametophytes and sporophytes; NCBI SRA: SRR10527930–SRR10527937). Gene expression levels were quantified as fragments per kilobase of transcript per million fragments mapped (FPKM), and expression heat maps were created using TBtools software based on log2 transformed FPKM values. Next, qRT-PCR was performed using established protocols from a previous study (Yu et al., 2020). We utilized the LightCycler^®^ 480 Real-Time PCR System to confirm the identity and expression of the selected genes. Ubiquitin-conjugating enzyme (UBC) genes were used as a reference (Gao et al., 2018). The primer sequences are listed in **Supplementary Table S1**. The comparative 2^(-ΔΔCt) method was used to determine the relative gene expression levels.

### 2.6 Expression profile analysis of *Ny. yezoensis bZIP* genes under nitrogen stress

To investigate the expression patterns of *NyybZIP* genes under nitrogen stress, a lab-cultured pure RZ *Ny. yezoensis* line was used in this study. Leafy gametophytes (thalli) of the *Ny. yezoensis* were maintained in 2 L aerated bottles with the Provasoli’s enrichment solution (PES) medium under the following conditions: 10°C with 60 μmol photons·m^−2^ s^−1^ and a 12 h:12 h light:dark (L:D) photoperiod, until the thallus length reached 5-8 cm. Then we set up the nitrogen-deficiency experiment consisted of two treatment groups: zero-nitrogen (N0) and control (C). The NaNO_3_ content in the Provasoli’s enrichment solution (PES) medium was as follows: 0 μM NaNO_3_ (N0) and 500 μM NaNO_3_ (C). All other cultivation conditions remained consistent across the treatment groups, and three biological replicates were used for each treatment group. After an eight-day cultivation period, the samples were collected in liquid nitrogen then sent to the company for RNA-seq data. And used DESeq2 (Love et al., 2014) to perform RNAs differential expression analysis, we identify DEGs (Fold Change > = 2 and FDR < 0.05). In addition, total RNA was extracted using the RNeasy Plant Mini Kit (OMEGA) according to the manufacturer’s instructions. Next, approximately 1 μg of the obtained total RNA was utilized for the synthesis of first-strand cDNA using the HiScript^®^ III RT SuperMix for qPCR (+gDNA wiper) Kit (Vazyme Biotech Co., Ltd., Nanjing, China). qRT–PCR was then performed using the primers listed in **Supplementary Table S1**.

## 3 Results

### 3.1 Identification of bZIPs in *Ny. yezoensis* and other red algae

**Figure 1:**
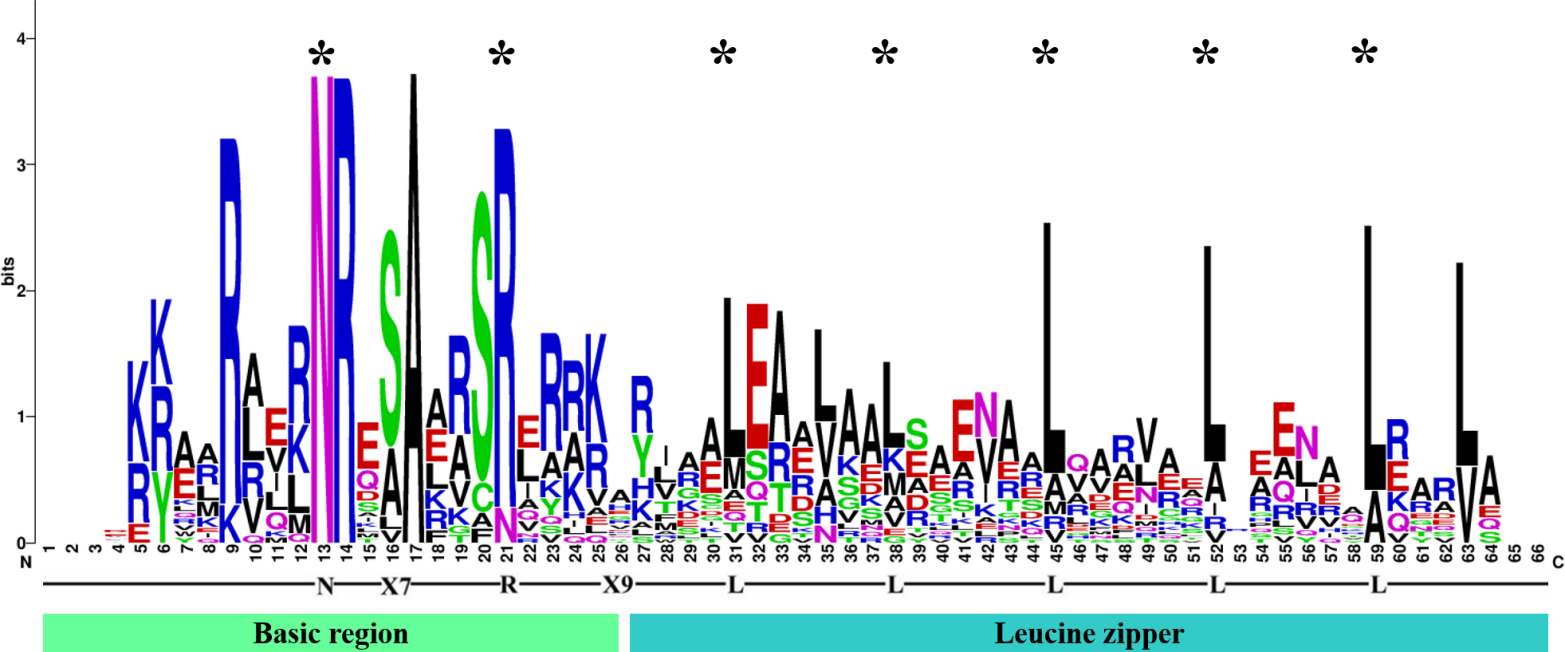
Visualization of multiple sequence alignment of the *Ny yezoensis* basic leucine zipper transcription factor family domains. The total height of the letter piles at each position indicates the conservation of the sequence at that position (measured in bits). The height of a single letter in the letter piles represents the relative frequency of the corresponding amino acid at that position. The symbol * means the conserved site.

**Figure 2:**
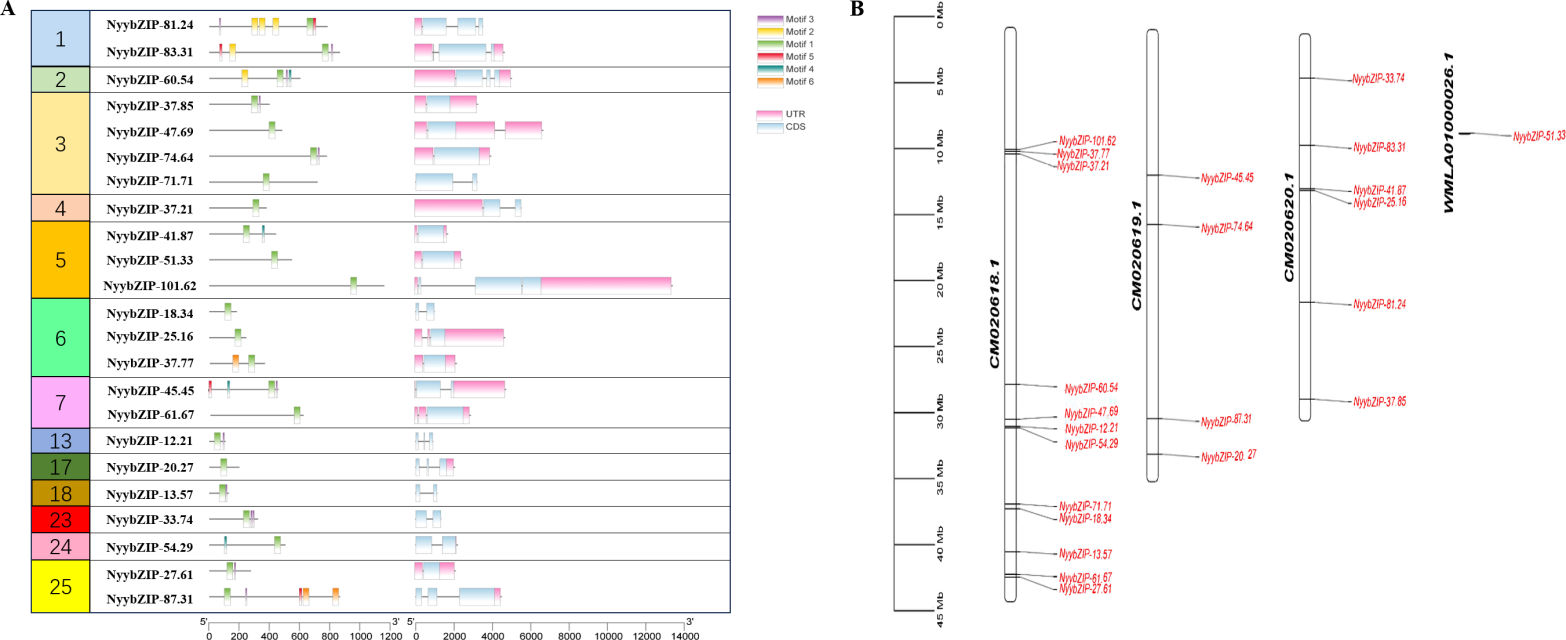
**(A)** Motifs and gene structures of the *NyybZIP* genes. **(B)** Chromosome distribution of the *Ny. yezoensis bZIP* genes. UTR, untranslated region; CDS, coding sequence.

To identify the *bZIP* genes in *Ny. yezoensis*, HMM, BLASTP, and PlnTFDB databases (http://plntfdb.bio.uni-potsdam.de/v3.0/) were used to identify and authenticate bZIP protein sequences in *Ny. yezoensis*. Next, Pfam, SMART, and CDD were used to determine the integrity of the bZIP domain. The amino acid positions of the conserved structural domains of bZIP were visualized by multiple sequence alignment of the protein sequences in *Ny yezoensis* bZIP family members (**Figure 1**). Evidence indicated that the bZIP domain consisted of a basic DNA-binding region and an adjacent leucine zipper structure. As shown in **Figure 1**, the basic DNA-binding region had an invariable N-X7-R/N motif and the adjacent leucine zipper structure created an amphipathic helix with heptapeptide repeat of Leucine (L) or related hydrophobic amino acid. Methionine, alanine, valine, etc. can replace highly conserved leucine residues in some cases. Such results of *Ny. yezoensis* bZIPs were consistent with those of previous studies in other plants (Jakoby et al., 2002; Dröge-Laser et al., 2018; Zhao et al., 2021). In total, 23 non-redundant genes were determined to be *Ny. yezoensis bZIP* genes and were named according to their molecular weight (**Table 1**). The following ranges were observed: gene size: 885–13383 bps; the number of introns: 0–2; number of amino acids residues: 108– 1159; isoelectric point (pI): 4.79–11.25; molecular weight (MW): 12.21–101.62 kDa; and instability index: 40.05–74.45. To investigate the functions and diversification of bZIP protein sequences in *Ny yezoensis*, we used the MEME software to predict their conserved motifs. Among them, motif1 was identified as the bZIP domain, which is present in all 23 NyybZIPs, whereas the other motifs lack specific annotation information (**Figure 2A**). Of the 23 *NyybZIP* genes identified, nine were intron-less, seven possess one intron, and seven possess two introns (**Table 1**; **Figure 2A**). Based on their chromosomal locations, 22 *NyybZIP* genes exhibited uneven distribution across the three nori chromosomes, and only one *NyybZIP* gene was mapped to *WMLA01000026.1* (**Figure 2B**). Specifically, *CM020618.1*, *CM020619.1*, and *CM020620.1* contained twelve, four, and six *NyybZIP* genes, respectively. Protein information of the *bZIP* genes in the other 14 red algae species is detailed in **Supplementary Table S2**.

**Table 1:** Gene characteristics of bZIP gene family.

Name	Accession number	Chromosomal locations	Location coordinates (5’-3’)	Gene length (bp)	Introns	Protein length (aa)	MW (kDa)	pI	Instability index	Subcellular localization
*NyybZIP-51.33*	KAK1857381.1	WMLA01000026.1	1-2414	2414	0	548	51.33	8.12	53.91	Extracell. Nucleus.
*NyybZIP-101.62*	KAK1858334.1	CM020618.1	9234338-9247720	13383	2	1159	101.62	9.74	62.95	Extracell.
*NyybZIP-37.77*	KAK1858362.1	CM020618.1	9395975-9398090	2116	0	369	37.77	8.36	55.16	Extracell. Nucleus.
*NyybZIP-37.21*	KAK1858394.1	CM020618.1	9583484-9588968	5485	1	381	37.21	10.54	61.79	Cytoplasm. Extracell. Nucleus.
*NyybZIP-60.54*	KAK1860595.1	CM020618.1	27034958-27039953	4996	2	604	60.54	11.25	74.45	Nucleus.
*NyybZIP-47.69*	KAK1860941.1	CM020618.1	29658296-29664928	6633	0	483	47.69	8.47	53.64	Nucleus.
*NyybZIP-12.21*	KAK1861020.1	CM020618.1	30229529-30230413	885	2	108	12.21	6.84	48.05	Nucleus.
*NyybZIP-54.29*	KAK1861032.1	CM020618.1	30328943-30331118	2176	1	505	54.29	10.26	56.75	Extracell. Mitochondrion. Nucleus.
*NyybZIP-71.71*	KAK1861704.1	CM020618.1	36105215-36108403	3189	1	718	71.71	4.79	60.35	Cytoplasm. Extracell. Nucleus.
*NyybZIP-18.34*	KAK1861742.1	CM020618.1	36455595-36456560	966	1	183	18.34	7.98	41.03	Endoplasmic reticulum. Extracell.
*NyybZIP-13.57*	KAK1862209.1	CM020618.1	39712897-39713997	1101	1	130	13.57	5.44	40.05	Extracell.
*NyybZIP-61.67*	KAK1862440.1	CM020618.1	41420379-41423232	2854	0	625	61.67	6.18	60.79	Endoplasmic reticulum.
*NyybZIP-27.61*	KAK1862464.1	CM020618.1	41621731-41623801	2071	0	276	27.61	4.98	41.74	Nucleus.
*NyybZIP-45.45*	KAK1864143.1	CM020619.1	11027149-11031846	4698	1	459	45.45	10.7	66.27	Nucleus.
*NyybZIP-74.64*	KAK1864633.1	CM020619.1	14765666-14769575	3910	0	779	74.64	9.55	59.89	Nucleus.
*NyybZIP-87.31*	KAK1866404.1	CM020619.1	29462426-29466896	4471	2	867	87.31	8.29	41.22	Cytoplasm.
*NyybZIP-20.27*	KAK1866701.1	CM020619.1	32156959-32158988	2030	2	200	20.27	9.95	53.61	Extracell.
*NyybZIP-33.74*	KAK1867359.1	CM020620.1	3346666-3347976	1311	1	323	33.74	8.8	54.76	Extracell. Nucleus.
*NyybZIP-83.31*	KAK1867951.1	CM020620.1	8437562-8442176	4615	2	866	83.31	9.63	66.66	Nucleus.
*NyybZIP-41.87*	KAK1868361.1	CM020620.1	11725390-11727050	1661	0	443	41.87	5.87	52.09	Extracell. Nucleus.
*NyybZIP-25.16*	KAK1868372.1	CM020620.1	11861594-11866234	4641	0	245	25.16	10.19	53.78	Extracell. Nucleus.
*NyybZIP-81.24*	KAK1869338.1	CM020620.1	20331938-20335433	3496	2	784	81.24	9.38	48.84	Endoplasmic reticulum. Extracell. Nucleus.
*NyybZIP-37.85*	KAK1870272.1	CM020620.1	27671737-27674965	3229	0	400	37.85	7.78	49.19	Nucleus.

### 3.2 Phylogenetic and motif analyses of *Ny. yezoensis* and other red algae *bZIP* gene families

**Figure 3:**
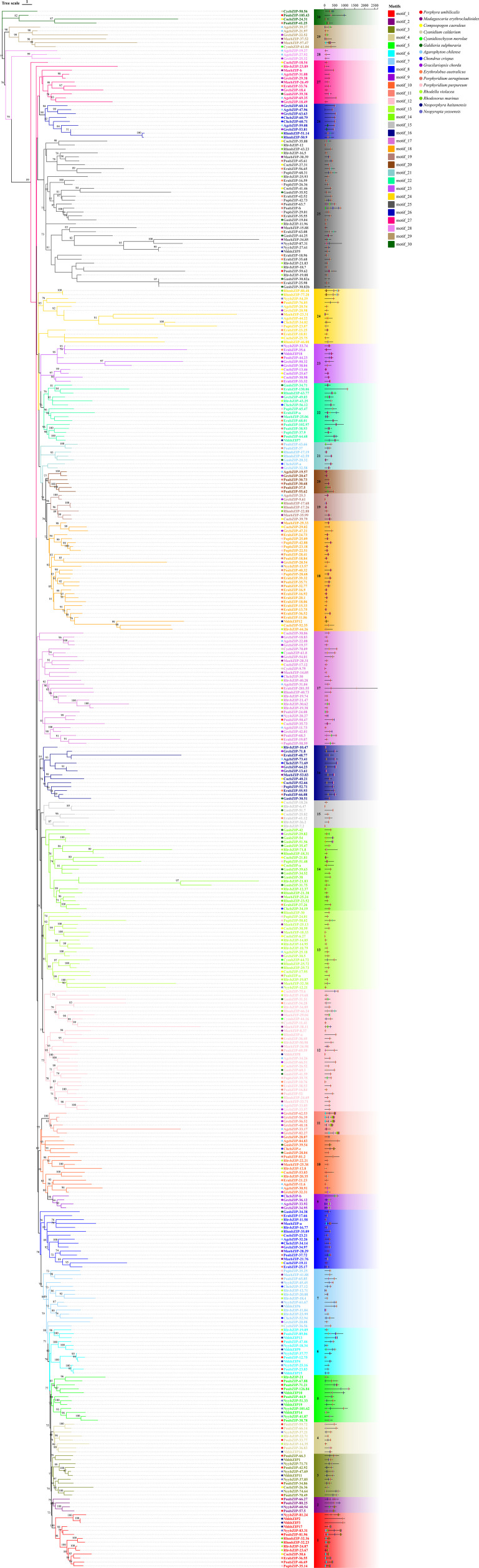
Phylogenetic relationship and conserved motifs of the bZIP proteins from 16 species of red algae. Different motifs are represented by different colored boxes. Conserved motifs were scanned with MEME.

To explore the evolutionary and classification relationships of the red algae bZIP families, we constructed a Maximum Likelihood (ML) phylogenetic tree using the bZIP domains of 16 types of red algae and 4 representative species of Viridiplantae bZIP protein sequences (**Supplementary Figure S1**). We found that bZIP protein sequences of bryophyte, green algae, and streptophyte algae could cluster with *A. thaliana*, and only a small portion of the red algae bZIP protein sequences could be classified into the *A. thaliana* bZIP groups; the majority of the red algae bZIP sequences formed distinct group, which we designated as Group R1 and R2. Among the NyybZIP protein sequences, only NyybZIP-18.34 and NyybZIP-20.27 were present in group M and classified with *A. thaliana*, whereas the other 21 sequences were distributed within Group R1 and R2. Therefore, we constructed a new phylogenetic tree using the complete sequences of bZIP proteins from 16 different species of red algae, which were classified based on the evolutionary relationships displayed in the constructed phylogenetic tree as well as the motif structures present (**Figure 3**). The bZIP proteins were divided into 30 groups (1–30), and phylogenetic analysis showed that NyybZIP-81.24 and NyybZIP-83.31 were in group 1 with motif 11; NyybZIP-60.54 was in group 2; NyybZIP-71.71, NyybZIP-47.69, and NyybZIP-74.64 were in group 3; NyybZIP-37.21 was in group 4; NyybZIP-41.87, NyybZIP-101.62, and NyybZIP-51.33 were in group 5; NyybZIP-18.34, NyybZIP-37.77, and NyybZIP-25.16 were in group 6; NyybZIP-45.45 and NyybZIP-61.67 were in group 7; NyybZIP-12.21 was in group 13; NyybZIP-20.27 was in group 17; NyybZIP-13.57 was in group 18; NyybZIP-33.74 was in group 23; NyybZIP-54.29 was in group 24; and NyybZIP-87.31 and NyybZIP-27.61 were in group 25.

**Figure 4:**
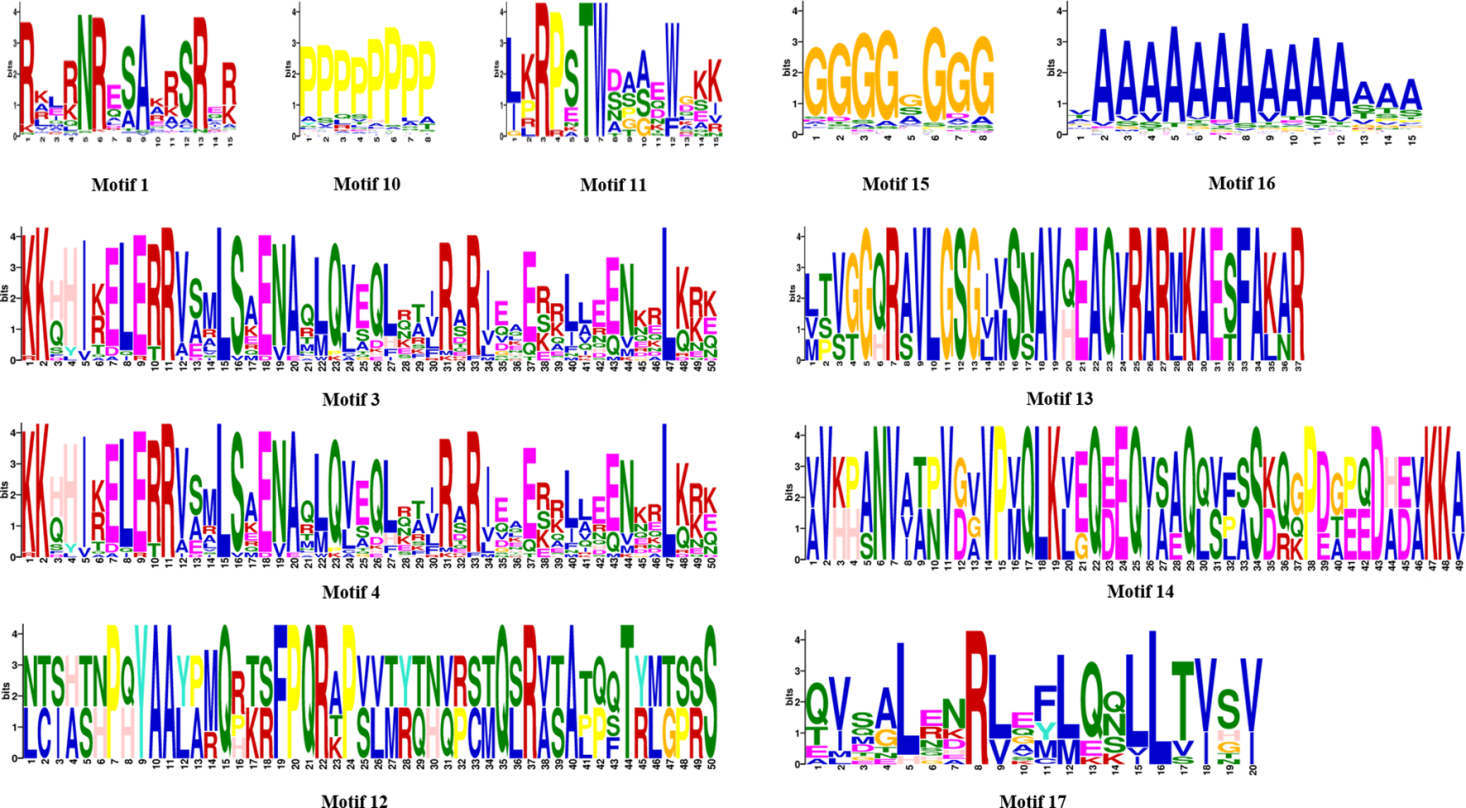
Discriminative motifs in the phylogenetic classification of *Ny. yezoensis* bZIP protein sequences.

In addition, phylogenetic analysis showed that motif 1 was the basic DNA-binding region of bZIP domain, motif 3 and motif 4 were the common Leucine zipper structure of bZIP domain found in red algae, and other motifs such as motifs 10, 15, and 16 were frequently observed in red algae bZIP protein sequences. Notably, due to their unique structural characteristics, motifs 11, 12, 13, 14, and 17 were employed as discriminative elements in the phylogenetic classification of red algae bZIP protein sequences (**Figure 4**). Many motifs exist in specific groups, which might be related to specific biological functions. Throughout the first evolutionary tree (**Supplementary Figure S1**), NyybZIPs did not cluster with the AtbZIP members, the similarity of bZIP proteins between *Ny. yezoensis* and *A. thaliana* showed low amino acid conservation which may be due to their relatively distant evolutionary relationship. In the second evolutionary tree (**Figure 3**), the *bZIP* genes of *Ny. yezoensis* showed good similarity with that of the other 15 red algae. And NyybZIPs clustered together with PoubZIPs and NhhbZIPs, which is consistent with the evolutionary relationships of red algae species.

### 3.3 Collinearity analysis of the *Ny. yezoensis bZIP* gene families

**Figure 5:**
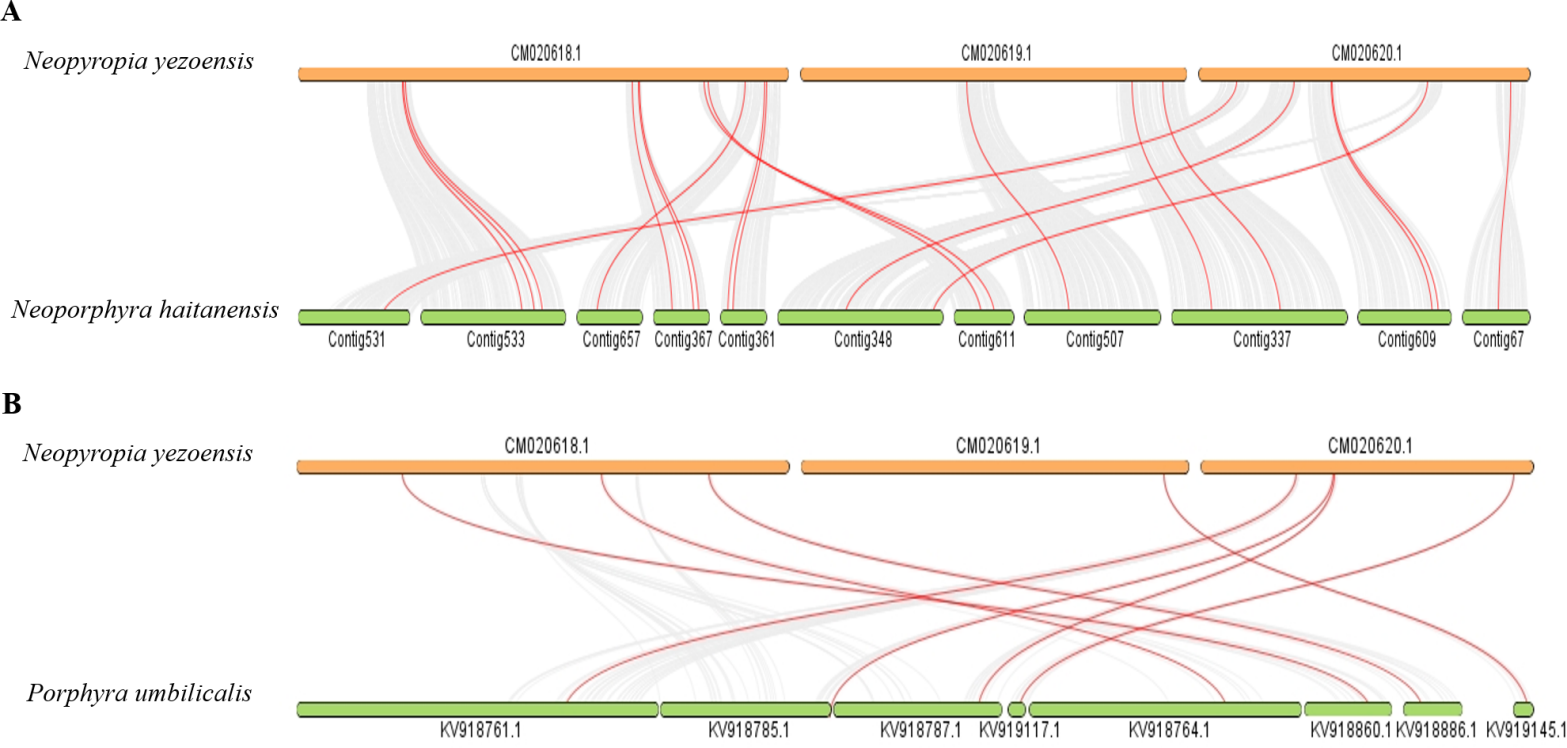
**(A)** Synteny analysis of the *bZIP* genes between *Ny. yezoensis* and *No. haitanensis*. **(B)** Synteny analysis of the *bZIP* genes between *Ny. yezoensis* and *P. umbilicalis*. The gray lines in the background indicate collinear blocks within the three red algal genomes. The red lines highlight syntenic *bZIP* gene pairs.

The One-Step MCScanX module within TBtools was used to identify and analyze tandem and segmental duplication events. And no tandem and segmental duplication events were detected in the *bZIP* gene family members of all these red algae species. Gene collinearity analysis was carried out between *Ny. yezoensis* and the other red algae with gene files and annotation files, the results showed that only the *bZIP* genes of *No. haitanensis* and *P. umbilicalis* have collinearity with the ones of *Ny. yezoensis* (**Figures 5A, B**). Twenty and eight *NyybZIP* genes were orthologous to the *bZIP* genes in *No. haitanensis* and *P. umbilicalis*, respectively. The ratio between the non-synonymous (Ka) and synonymous (Ks) has been widely used as an index for measuring the strength and direction of selection pressure. Ka/Ks > 1 means positive selection, Ka/Ks < 1 means purifying selection, and Ka/Ks = 1 means neutral selection. We found that all identified collinear gene pairs exhibited Ka/Ks ratio values well below one (**Supplementary Table S3**), confirming strong purifying selection during the evolution of the *bZIP* gene family in the *Bangiaceae* family. This observation aligns with our findings from the unrooted phylogenetic tree (**Figure 3**), suggesting that these genes may play important roles in the evolution of the red algae *bZIP* gene family.

### 3.4 Cis-regulatory element analysis of the *Ny. yezoensis bZIP* gene families

**Figure 6:**
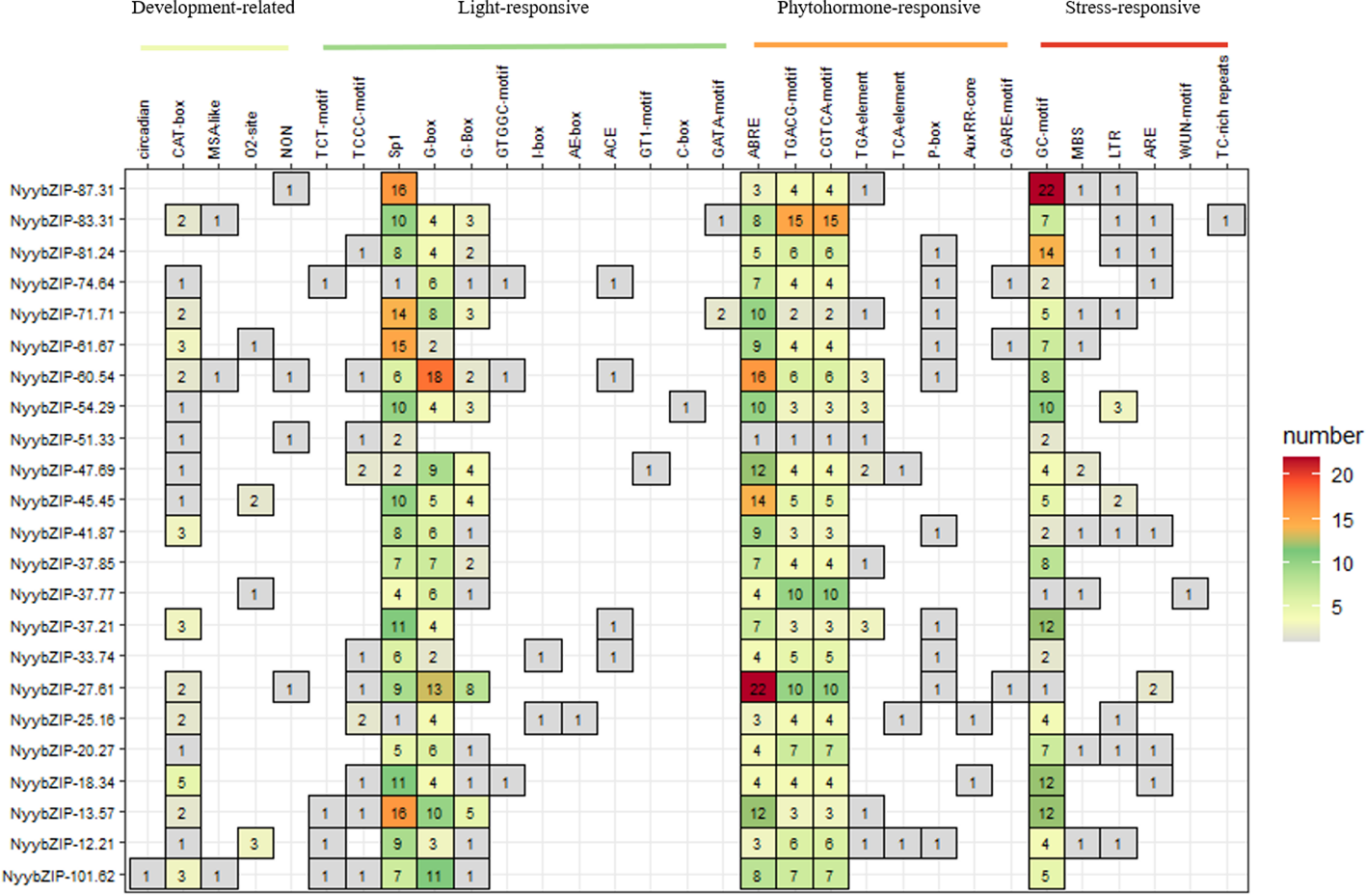
Analysis of 2 kb upstream cis-acting elements found in red algae *bZIP* genes. The different colors and numbers of the grid indicate the numbers of different promoter elements.

Cis-regulatory elements play critical roles in the transcriptional regulation of gene expression and control various biological processes (Yamaguchi-Shinozaki and Shinozaki, 2005). To further explore the regulatory mechanisms of the *bZIP* genes in *Ny. yezoensis* growth, development, and stress responses, we analyzed cis-elements within the promoter sequences (2 kb) of 23 *Ny. yezoensis bZIP* genes by the PlantCARE database. Our analysis identified four main categories of cis-elements: development-related, light-responsive, phytohormone-responsive, and stress-responsive (**Figure 6**). Among these categories, phytohormone-responsive elements were the most abundant (41.77%), and included ABRE (involved in abscisic acid responsiveness), AuxRR-core and the TGA-element (involved in auxin responsiveness), CGTCA- and TGACG-motifs (involved in MeJA-responsiveness), and the GARE-motif and P-box (involved in gibberellin-responsiveness). Light-responsive elements were the second most abundant (36.38%), followed by the stress-responsive elements (17.18%). Stress-responsive elements included ARE (essential for anaerobic induction), the GC-motif (involved in anoxic-specific inducibility), LTR (involved in low-temperature responsiveness), MBS (involved in drought-inducibility), and TC-rich repeats (involved in defense and stress responsiveness). Development-related elements (4.66%) were the least abundant and included circadian (involved in circadian control), CAT-box (related to meristem expression), MSA-like (involved in cell cycle regulation), and O2-site (cis-acting regulatory element involved in zein metabolism regulation). Overall, analysis of cis-acting elements showed that *NyybZIP* genes have diverse functions in the transcriptional regulation of gene expression and control various biological processes.

### 3.5 *NyybZIP* gene expression patterns at different life stages

**Figure 7:**
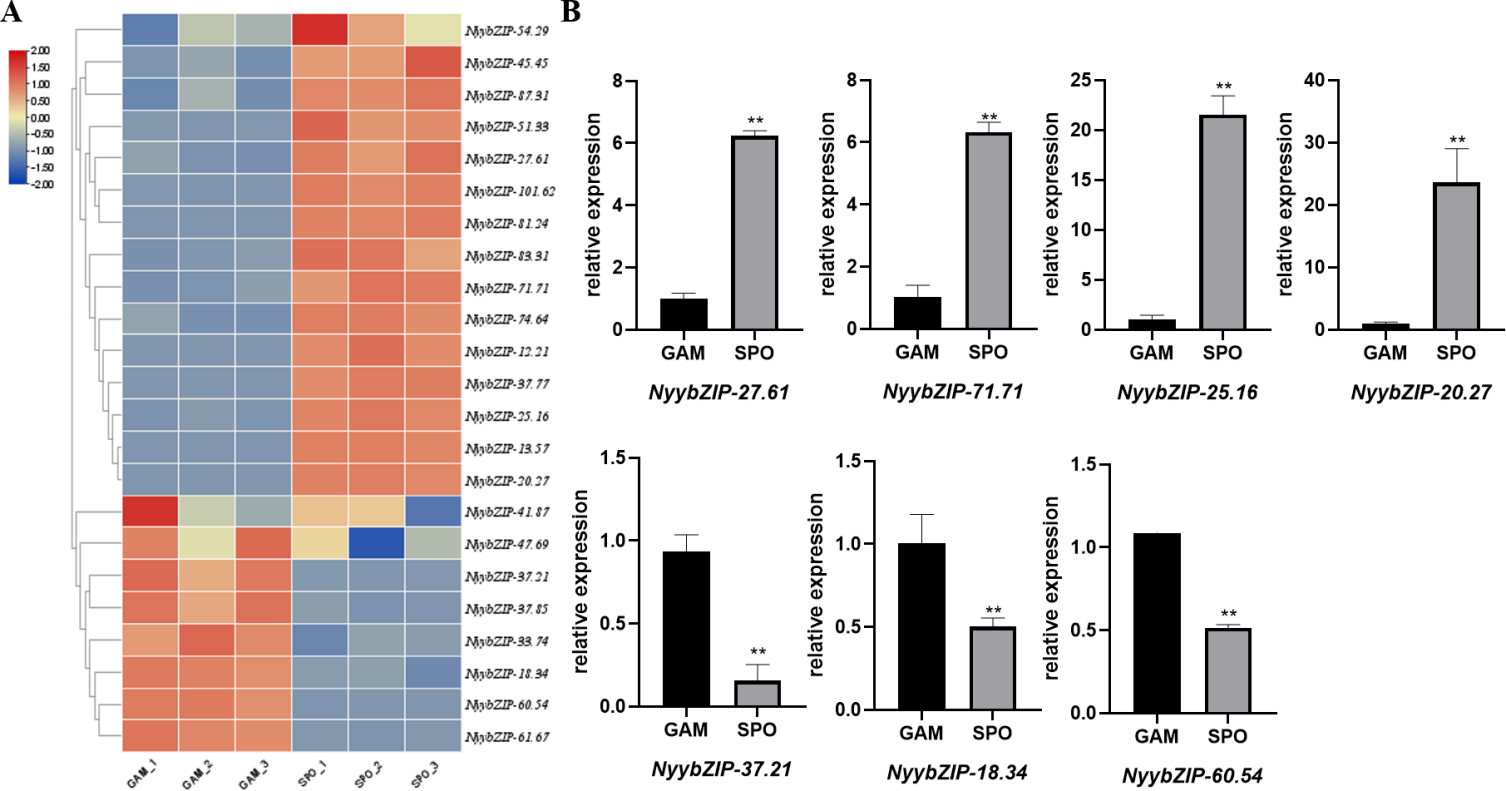
**(A)** *NyybZIP* gene expression patterns at different life stages. **(B)** Expression analysis of seven representative genes from the *Ny. yezoensis* bZIP family at different life stages. GAM, gametophytic haploid blades; SPO, sporophytic diploid filaments; **indicates a significant difference of p<0.01.

*Ny. yezoensis* exhibits a sophisticated life cycle characterized by recurrent alternation between two distinct multicellular phases: gametophytic haploid blades (GAM) and sporophytic diploid filaments (SPO). **Figure 7A** illustrates the expression patterns of the 23 *NyybZIP* genes across these two life cycle stages. *NyybZIP* genes were expressed in both the sporophyte and gametophyte stages and demonstrated different expression levels. *NyybZIP-41.87*, *NyybZIP-47.69*, *NyybZIP-37.21*, *NyybZIP-37.85*, *NyybZIP-33.74*, *NyybZIP-18.34*, *NyybZIP-60.54*, and *NyybZIP-61.67* exhibited higher expression levels in gametophytes than in sporophytes. In contrast, other *NyybZIP* genes exhibited lower expression levels in gametophytes than in sporophytes. These findings indicate distinct expression of *bZIP* genes in *Ny. yezoensis* across different stages of growth and development, suggesting specific regulatory roles where genes highly expressed in one stage may undergo significant changes in expression levels during another phase. These observations suggest that various *bZIP* genes play critical roles in the growth and development of *Ny. yezoensis* throughout its life cycle stages.

Next, we designed primers for seven *NyybZIPs* and performed quantitative real-time polymerase chain reaction (qRT–PCR) analysis (**Figure 7B**). *Nyy-27.81*, *Nyy-71.71*, *Nyy-25.16*, and *Nyy-20.27* were highly expressed in SPO and exhibited lower levels in GAM. Conversely, *Nyy-37.21*, *Nyy-18.34*, and *Nyy-60.54* exhibited low expression in SPO but were highly expressed in GAM. The qRT-PCR results validate the authenticity and reliability of the *NyybZIP* RNA-seq data in the two life cycle stages and provide a foundation for further investigation into the regulatory mechanisms underlying the differential expression patterns of *bZIP* genes in *Ny. yezoensis* at different life stages.

### 3.6 *NyybZIP* gene expression profile analysis under nitrogen stress

**Figure 8:**
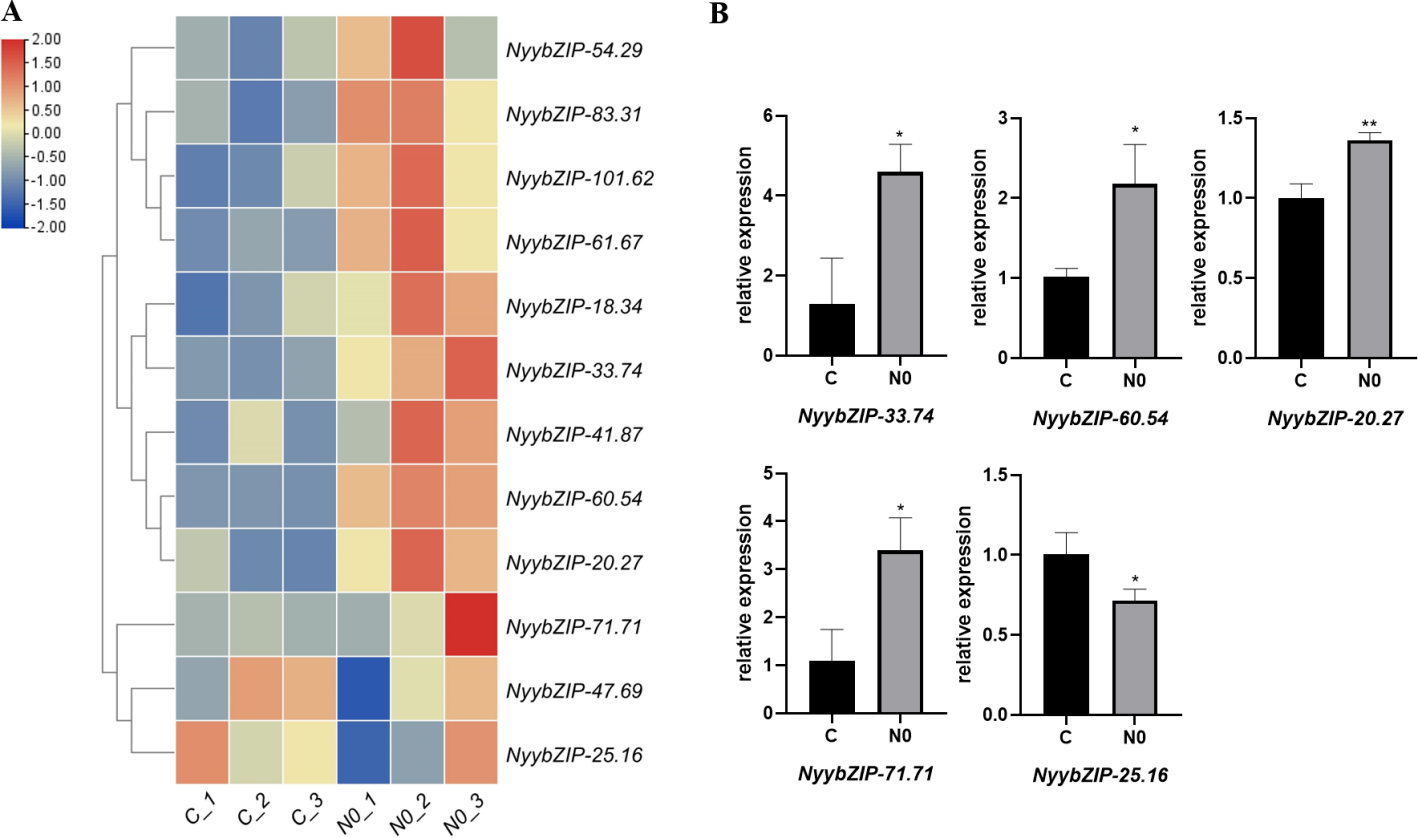
**(A)** The significant differential expression patterns of select *NyybZIP* genes under nitrogen stress. **(B)** Expression analysis of five representative genes from the *Ny. yezoensis* bZIP family under nitrogen stress. N0, zero nitrogen group; C, control group. ** and * indicate significant differences of p<0.01 and p<0.05, respectively.

To explore the impact of nitrogen stress on *Ny. yezoensis*, we investigated the differential expression patterns of *NyybZIP* family members in response to abiotic stress. Based on the RNA-seq data from nitrogen stress treatments, we identified a subset of *NyybZIP* genes that exhibited differential expression levels. A heat map was constructed to visualize these findings (**Figure 8A**). Most of the *NyybZIP* genes exhibited higher expression levels under low nitrogen stress, with *NyybZIP-54.29*, *NyybZIP-83.31, NyybZIP-101.62, NyybZIP-61.67, NyybZIP-41.87, NyybZIP-18.34*, *NyybZIP-33.74, NyybZIP-60.54*, *NyybZIP-71.71*, and *NyybZIP-20.27* demonstrating higher expression levels in the N0 group compared to those of the C group. However, *NyybZIP-47.69* and *NyybZIP-25.16* had lower expression levels in the N0 group compared to those in the C group. These results suggest that *NyybZIP* genes can exhibit responsive expression under nitrogen stress, which is consistent with observations in previous studies on *A. thaliana* (Sun et al., 2012; Alvarez et al., 2014).

Next, we designed primers for five representative *NyybZIPs* and conducted qRT-PCR analysis (**Figure 8B**). Our results revealed that *Nyy-33.74*, *Nyy-60.54*, *Nyy-20.27*, and *Nyy-71.71* were highly expressed in the N0 group and exhibited lower expression levels in the C group. However, *Nyy-25.16* exhibited low expression in the N0 group but was highly expressed in the C group. The qRT-PCR results validated the RNA-seq data, suggesting that under low nitrogen stress conditions, *NyybZIP* genes can respond to stress by altering expression levels. These results provide a foundation for further investigations into the potential roles of these genes under nitrogen stress and their associated functions.

## 4 Discussion

### 4.1 Identification and characterization of the *Ny. yezoensis bZIP* gene families

*Ny. yezoensis*, a key species in the commercial cultivation of edible marine macroalgae, has a sophisticated life cycle (Blouin et al., 2011). Like other plants, *Ny. yezoensis* is subjected to abiotic stresses, including nitrogen. The *bZIP* genes play an important role in the regulation of growth, development, and abiotic stress responses (Landschulz et al., 1988). The number of bZIP family members varies among different plant species, including 78 members in *A. thaliana* (Dröge-Laser et al., 2018), 19 in *No. haitanensis* (Wang et al., 2023), and 92 in rice (Corrêa et al., 2008). Despite extensive research on bZIP families in numerous plant species, relatively few studies have characterized bZIP families in red algae. From previous research in *No. haitanensis* (Wang et al., 2023), we found that the bZIP protein sequences of some red algae did not fit well into the classification established for *A. thaliana* (Dröge-Laser et al., 2018), a considerable fraction of them cannot be classified effectively. Moreover, the red algae species selected in the article are limited. So, in the present study, we selected the annotation results, gene sequence and protein sequence uploaded by *Ny. yezoensis* in NCBI as the data support, and gathered protein information from NCBI and EukProt (Brawley et al., 2017) from 14 representative species covering all seven classes of Rhodophyta. We identified 23 bZIP family members in *Ny. yezoensis* and found that the number of bZIP family members in these red algae ranged from 4 to 45. This count is notably lower when compared to *A. thaliana* and other plants, which is consistent with previous research (Bhattacharya et al., 2018). We observed no direct correlation between the size of red algal genomes and the number of bZIP family members.

In our analysis of bZIP family genes in *Ny. yezoensis*, we investigated the chromosome locations, number of introns, protein lengths, molecular weights, pI values, instability indices, and subcellular localizations. Our findings revealed that the number of introns ranged from 0 to 2, and the gene structure was relatively simple, which is consistent with observations in *Malus domestica* (Zhao et al., 2016). The presence of absence of introns has been linked to the upregulation of genes in response to diverse stressors (Jeffares et al., 2008), suggesting that the intron number plays a significant role in the diversification within multigene families (Wang et al., 2015). Genes with fewer introns are often associated with rapid activation in response to various stress conditions (Jeffares et al., 2008). The instability index estimates protein stability as a predictive measure, where values less than 40 generally indicate stable proteins (Gasteiger et al., 2005). Interestingly, our results showed that the instability indices of all *Ny. yezoensis* bZIPs were greater than 40, indicating that they were unstable proteins. This instability is regarded as a universal characteristic of stress-responsive proteins (Gao et al., 2022). However, the subcellular localization of proteins is closely related to their function, and proteins can only perform their proper functions at specific subcellular sites (Wang et al., 2014). Taken together, our results provide a reference for further investigation into the functional aspects of the bZIP protein family in *Ny. yezoensis*.

### 4.2 Phylogenetic classification of red algae bZIPs

We also investigated red algae bZIPs to classify and analyze the origin of their gene families. In *T. aestivum*, *Glycine max* and other plants, classification standards often use *A. thaliana* bZIP proteins based on the homology of their basic region, and additional conserved motifs have been proposed (Dröge-Laser et al., 2018). However, research on bZIP proteins in *No. haitanensis* (Wang et al., 2023) and our study found that most sequences of red algae bZIPs did not align with the classification system based on *A. thaliana*. This situation may be due to the early separation of the red algae with Viridiplantae. Thus, we classified bZIP protein sequences from 16 species of red algae into 30 groups using phylogenetic and motif analyses and provides a basis for the classification of *Ny. yezoensis*. However, owing to the lack of functional studies related to bZIP proteins in red algae, we can only conduct theoretical classification based on the currently available red algae protein sequence data. Nonetheless, this study can serve as a reference for future research on bZIP proteins in red algae and contribute to the investigation of their functions and roles.

### 4.3 Gene replication and collinearity analyses of the *Ny. yezoensis* bZIPs

Research indicates that genomic and gene duplication events play significant roles in plant evolution (Flagel and Wendel, 2009). Tandem and segmental duplications have also been frequently observed in the *bZIP* family genes within both *M. domestica* (Wang et al., 2021) and *P. pyrifolia* (Ma et al., 2021), and have contributed to the expansion and diversification of gene families. However, in *Ny. yezoensis* and other red algae, tandem duplications and segmental duplications were not detected, which is consistent with studies conducted on the red algae species *No. haitanensis* (Wang et al., 2023). Furthermore, we analyzed the collinearity between the *bZIP* genes of *Ny. yezoensis* and other red algae. The results proved that the *bZIP* genes of *Ny. yezoensis* had collinear gene pairs with *No. haitanensis* and *P. umbilicalis*. These three species had relatively close evolutionary relationships. And the calculated values of the Ka/Ks ratio for such gene pairs were less than 1, indicating that the *bZIP* gene family in these species likely underwent strong purifying selection pressure during evolution.

### 4.4 Expression and potential functions of the *Ny. yezoensis* bZIP gene families

Cis-regulatory elements play critical roles in the transcriptional regulation of gene expression and control various biological processes (Yamaguchi-Shinozaki and Shinozaki, 2005), making them essential tools for exploring the functions of bZIP proteins across numerous species (Hsieh et al., 2010; Wang et al., 2017). For example, under ABA and abiotic stress treatments, *A. thaliana* and *Solanum lycopersicum* plants exhibit overexpression of SlAREB, which regulates genes associated with stress responses, and overexpression of ABP9 in cotton results in increased sensitivity to exogenous ABA at seed germination, root growth, stomatal aperture, and stomatal density. All plant genes using the ABRE system require bZIP proteins for expression (Liao et al., 2008). In our study, ABRE was identified in all of the *NyybZIP* gene promoter sequences, suggesting that ABRE-mediated regulation may contribute to the involvement of *NyybZIP* genes in response to nitrogen stress. Previous articles in *A. thaliana* found that different *bZIP* genes affected the growth and development of different tissues (Murmu et al., 2010; Maier et al., 2011; Iglesias-Fernández et al., 2013). And in this study, we discovered that all *NyybZIP* genes displayed distinct expression levels at different life stages. So, we thought that these genes are involved in *Ny. yezoensis* growth and development.

Research has shown that nitrogen deficiency had a great impact on plant yield and quality. At the same time, nitrogen played an equally important role in *Ny. yezoensis*. When the cultivation area was short of nitrogen, severe thallus discoloration (“iroochi”) would occur, resulting in lower quality of *Ny. yezoensis* (Oyama et al., 2008). Based on *A. thaliana* research, bZIP protein sequences have been shown to play significant roles in nitrate stress, such as nitrate uptake, and nitrate responses (Sun et al., 2012). In *A. thaliana* roots, bZIP transcription factor mRNAs accumulated strongly after nitrate treatment, and regulated the expression of nitrate transporter genes *NRT2.1* and *NRT2.2* (Alvarez et al., 2014). Therefore, bZIP transcription factor played an important role in nitrogen absorption and transport Additional plant studies have demonstrated that multiple bZIP transcription factors are associated with nitrogen stress responses. For example, in *G. max*, the overexpression of *GmbZIP44*, *GmbZIP62*, and *GmbZIP78* can notably increase nitrogen stress tolerance and they were negative regulators of ABA signaling and function in stress (Liao et al., 2008). In *T. aestivum*, lower the expression of *TabZIP60* by RNA interference can increase NADH-dependent glutamate synthase (NADH-GOGAT) activity, N uptake and so on, while overexpression of *TabZIP60* had the opposite effects (Yang et al., 2019). However, the role of bZIP sequences in *Ny. yezoensis* needs to be further explored. In the present study, under nitrogen deficiency conditions, there were differences in gene expression levels, which was consistent with previous studies in *A. thaliana* and other plants. So, we thought that *NyybZIP* genes may play a significant role in nitrogen deficiency response of *Ny. yezoensis*. However, additional studies are required to elucidate the roles of specific genes.

## 5 Conclusions

In the present study, we identified 23 *bZIP* genes in *Ny. yezoensis* and performed a comprehensive analysis of *NyybZIP* genes based on gene structures, conserved motifs, phylogenetic relationships and so on. Then, they were identified and sorted into 13 out of 30 groups, which were classified based on the bZIPs of *Ny. yezoensis* and 15 other red algae species. Phylogenetic analysis revealed that *bZIP* genes may have a complex evolutionary pattern in red algae. In addition, cross-species collinearity analysis indicated that the *bZIP* genes in *Ny. yezoensis*, *No. haitanensis* and *P. umbilicalis* are highly evolutionarily conserved. Finally, we identified four main categories of cis-elements, including development-related, light-responsive, phytohormone-responsive and stress-responsive promoter sequences in *NyybZIP* genes and also explored the expression profiles of *NyybZIP* genes at different life stages and under nitrogen stress using RNA-seq data and performed qRT–PCR analysis of these genes. This study aids in developing a more thorough understanding of *NyybZIP* genes and creates a foundation for future functional characterization efforts. In addition, our study provides new insights into the classification of *bZIP* gene families in red algae and contributes to our understanding of the adaptability of *Ny. yezoensis* to nitrogen stress and the significant differences in gametophytic and sporophytic stages.

## Data Availability

To investigate the expression patterns of NyybZIP genes under nitrogen stress, the transcriptome data has deposited in the SRA database of NCBl (accession number: SRR30899373– SRR30899378).

## Supplementary material

Supplementary Figure S1 A Maximum Likelihood (ML) phylogenetic tree using the bZIP domains of 16 types of red algae and 4 representative species of Viridiplantae.
