# Supplementary figures and images for: Genome-wide analysis and expression profile of the bZIP gene family in Neopyropia yezoensis
Source: Front Plant Sci. 2024 Oct 21;15:1461922. doi: 10.3389/fpls.2024.1461922 (PMC11533322; doi:10.3389/fpls.2024.1461922)

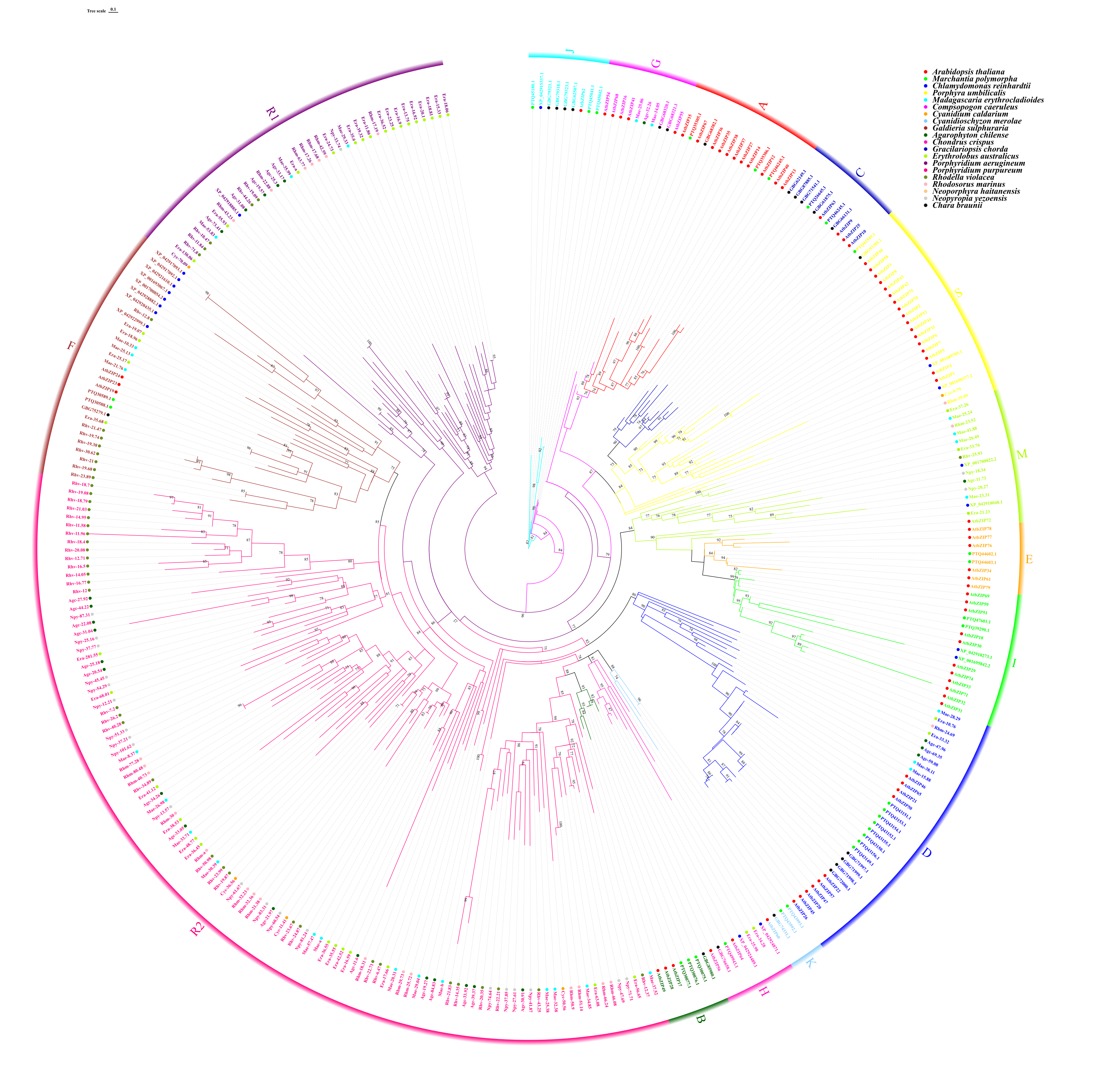

Supplement: Supplementary Figure S1 — A Maximum Likelihood (ML) phylogenetic tree using the bZIP domains of 16 types of red algae and 4 representative species of Viridiplantae. [file Image1.tif]
